# Optimal Skeletal Muscle Mass Index Cut-Off Values for Presarcopenia Evaluated by Computed Tomography against Dual-Energy X-ray Absorptiometry in Patients with Chronic Liver Disease

**DOI:** 10.3390/jcm10071419

**Published:** 2021-04-01

**Authors:** Kazuki Ohashi, Toru Ishikawa, Asami Hoshii, Tamaki Hokari, Hirohito Noguchi, Mitsuyuki Suzuki, Hiroshi Hirosawa, Michitaka Imai, Yuta Mitobe, Toshiaki Yoshida

**Affiliations:** 1 Department of Nursing, Sapporo University of Health Sciences, Hokkaido 007-0894, Japan; ohashi@sapporo-hokeniryou-u.ac.jp; 2 Department of Gastroenterology and Hepatology, Saiseikai Niigata Hospital, Niigata 950-1104, Japan; michitaka7supernova1979@gmail.com (M.I.); toshi@ngt.saiseikai.or.jp (T.Y.); 3 Department of Medical Radiology, Saiseikai Niigata Hospital, Niigata 950-1104, Japan; xray.hoshii@ngt.saiseikai.or.jp (A.H.); miuratama69@yahoo.co.jp (T.H.); 4 Department of Nursing, Saiseikai Niigata Hospital, Niigata 950-1104, Japan; naishikyo@ngt.saiseikai.or.jp; 5 Department of Pharmacology, Saiseikai Niigata Hospital, Niigata 950-1104, Japan; suzukimitsuyuki32@gmail.com; 6 Department of Clinical Engineering, Saiseikai Niigata Hospital, Niigata 950-1104, Japan; h.hirosawa@ngt.saiseikai.or.jp; 7 Graduate School of Nursing, International University of Health and Welfare, Tokyo 107-8402, Japan; yuta.mitobe@gmail.com

**Keywords:** sarcopenia, skeletal muscle mass, dual-energy X-ray absorptiometry, computed tomography

## Abstract

Although dual-energy X-ray absorptiometry (DXA) and body impedance analysis are commonly used to measure skeletal muscle mass (SMM), a computed tomography (CT) scan is preferred in clinical practice. We aimed to propose the cut-off values of skeletal muscle mass index (SMI) calculated using CT scans, using DXA as the reference method. We retrospectively assessed 589 patients with chronic liver disease. The SMI was assessed using appendicular SMM by DXA and total muscle area at the level of the third lumbar vertebra (L3) calculated by CT. The cut-off value was determined with reference to the Asian Working Group for Sarcopenia criteria. DXA identified 251 (42.6%) patients as having presarcopenia. In men, the cut-off value of SMI for presarcopenia was determined to be 45.471 cm^2^/m^2^, with an area under the curve (AUC) of 0.863 (95% confidence interval (CI): 0.823 to 0.903), and in women, this value was determined to be 35.170 cm^2^/m^2^, with an AUC of 0.846 (95% CI: 0.800 to 0.892). Cohen’s kappa coefficient was 0.575 (95% CI: 0.485–0.665) in men and 0.539 (95% CI: 0.438–0.639) in women.

## 1. Introduction

Sarcopenia, defined as decreases in muscle mass, strength, and physical function, is an important cause of functional decline associated with aging and chronic illness [1,2,3]. Presarcopenia is defined as the loss of muscle mass by European Working on Sarcopenia in Older People [2]. This condition can negatively influence clinical outcomes in patients with chronic liver disease (CLD) [4,5,6]. Moreover, presarcopenia has been associated with poor prognosis in patients with liver cirrhosis (LC) and hepatocellular carcinoma (HCC) [7,8], and sarcopenia impairs outcomes in patients undergoing liver transplantations [9]. In 2016, the Japanese Society of Hepatology (JSH) published diagnostic criteria for sarcopenia suitable for use in patients with liver disease [10]. These criteria diagnose sarcopenia based on grip strength and skeletal muscle mass index (SMI). Measurement methods of grip strength and SMI were decided based on ease of use in clinical practice. The grip strength cut-off values are <26 kg for men and <18 kg for women and SMI is calculated by determining the skeletal muscle area (SMA) at the third lumbar vertebra (L3) level using computed tomography (CT) or the skeletal muscle mass (SMM) by body impedance analysis (BIA) divided by the square of height (cm^2^/m^2^ or kg/m^2^). The JSH cut-off values for SMI calculated by CT are ≤42.0 cm^2^/m^2^ for men and ≤38.0 cm^2^/m^2^ for women [10]. The cut-off values of SMI evaluated by CT were set based on the cut-off values of SMI evaluated by BIA according to the Asian Working Group for Sarcopenia (AWGS). The AWGS criteria are widely used in Asia [1,11]. According to these criteria, dual-energy X-ray absorptiometry (DXA) or BIA should be used for measuring SMM. SMI determined by CT and appendicular skeletal muscle mass index (ASMI; kg/m^2^) determined by DXA have correlated in previous studies [12,13,14]. However, these studies did not focus on Asian people and did not have optimal cut-off values for SMI determined by CT. CT is often used in japan for examining patients with CLD as screening for HCC [10]. Namely, an appropriate cutoff value of SMI by CT enables objective and retrospective verification. Moreover, AWGS criteria do not include cut-off values for SMI measured by CT. Adapting the AWGS criteria could improve the reliability and validity of the cut-off value for SMI by CT. Furthermore, the development of a new cut-off value could allow for the simultaneous estimation of the risk of presarcopenia and that of HCC for patients with CLD. Therefore, in this study, we analyzed the cut-off values of SMI by CT in patients with CLD and proposed optimized cut-off values of SMI by CT, using ASMI by DXA as the reference method. 

## 2. Materials and Methods

### 2.1. Patients

In this single-center, cross-sectional retrospective study, we included outpatients who presented with CLD between October 2015 and November 2020 at the Department of Gastroenterology, Saiseikai Niigata Hospital (Niigata, Japan). Consecutive data were included in our analysis. Patients who underwent CT scans and DXA within a week were included in this study. CT scan was routinely conducted in patients with CLD for screening or surveillance of HCC. DXA was routinely conducted in patients for assessing hepatic osteodystrophy. Patients were excluded if they had existing metal in their bodies. Informed consent was obtained on our website; those who opted out were excluded. The study protocol was approved by the ethics committee of the Saiseikai Niigata Hospital (approval number E18-18) and carried out in accordance with the Helsinki Declaration.

### 2.2. Evaluation of SMM and Diagnosis of Presarcopenia

The SMM of all four extremities was calculated to obtain appendicular skeletal muscle mass (ASM) (kg) using DXA, Discovery QDR SERIES Wi^®^ (Hologic Inc., Waltham, MA, USA). Then, the value of ASM was divided by the square of the height in meters to obtain the ASMI (kg/m^2^). The cross-sectional SMA (cm^2^) at L3 was measured using a CT scan, Aquilion ONE^®^ (Canon Medical Systems Co., Tochigi, Japan), using the SYNAPSE VINCENT^®^ medical imaging system (Fujifilm Medical Co., Ltd., Tokyo, Japan) to trace the area—more specifically, using the Hounsfield unit (HU) range for SMA absorbing X-rays (−29, +150), and the cross-sectional area of the above muscles was semi-automatically traced. To obtain the SMI, the cross-sectional SMA (cm^2^) was divided by the square of the height (m^2^). Presarcopenia was defined as loss of skeletal muscle mass and diagnosed in this study according to AWGS criteria [1] and JSH criteria [10]: ASMI < 7.0 kg/m^2^ for men and <5.4 kg/m^2^ for women; SMI ≤ 42 cm^2^/m^2^ for men and ≤38 cm^2^/m^2^ for women. 

### 2.3. Clinical and Laboratory Assessment

Data on the following characteristics were collected: sex, age, body mass index (kg/m^2^), etiology of liver disease, and biochemical data (platelet count, serum albumin, alanine aminotransferase, aspartate aminotransferase). In addition, based on the biochemical data collected, the albumin–bilirubin (ALBI) score, ALBI grade [15,16] and fibrosis-4 index (FIB-4) [17,18,19] were calculated for the estimation of hepatic reserve and disease severity.

### 2.4. Statistical Analysis

Continuous variables are presented as mean values ± standard deviations (SD) and were compared using Welch’s *t*-test. Categorical variables and nominal variables were presented as absolute numbers and frequencies, and comparisons were performed using Fisher’s exact test. The correlation between SMI by CT and ASMI by DXA was analyzed using Pearson’s correlation coefficient. Z-scores of SMI and ASMI were calculated for each sex, based on the means of SMI and ASMI and the corresponding SD from our study sample. The Z-score made it possible to compare SMI by CT and ASMI by DXA. The reliability of SMI against ASMI was evaluated using Bland–Altman plots [14,20]. The normality of the mean differences between all Z-scores was examined using the Shapiro–Wilk test and presented in a histogram. The plots show the mean differences (ASMI minus SMI in each Z-score) and the means of both Z-scores [20]. The biases and the corresponding limits of agreement (±1.96 SD) of the mean difference were calculated [14]. The cut-off values of SMI for presarcopenia using CT were calculated by receiver-operating characteristic curve (ROC) analysis with the Youden index [21]. Against the reference ASMI by DXA, sensitivity, specificity, positive and negative predictive values, positive and negative likelihood ratios, and accuracy were calculated. Data were analyzed using R version 4.0.3 (R Foundation for Statistical Computing, Vienna, Austria) [22] and EZR ver. 1.42 (Saitama Medical Center, Jichi Medical University, Saitama, Japan) [23]. *p* values < 0.05 indicate statistical significance.

## 3. Results

A total of 589 patients with CLD (312 men and 277 women) were included in this study. The mean age (±SD) was 63.15 (±13.69) years for men and 66.22 (±12.79) years for women. The mean body mass index was 23.33 (±3.9); 123 patients (20.9%) had HCC, and 164 patients (27.8%) had cirrhosis. We found that 251 (42.6%) patients were diagnosed with presarcopenia according to AWGS criteria. According to JSH criteria, 278 (47.2%) patients were diagnosed with presarcopenia. There were more women with presarcopenia than men. Whole-body bone mineral density was 1.07 (0.12) in men and 0.91 (0.12) in women (Table 1).

Data are expressed as means (±SD) or as *n* (%). Statistical analysis was carried out using Fisher’s exact test or Welch’s *t*-test, as appropriate.

AWGS: Asia Working Group for sarcopenia, JSH: Japan society of hepatology, CT: computed tomography, DXA: dual-energy X-ray absorptiometry, 

HBV: hepatitis B virus, HCV: hepatitis C virus, NASH: nonalcoholic steatohepatitis, PBC: primary biliary cirrhosis, AIH: autoimmune hepatitis, 

ALBI: albumin–bilirubin, FIB-4: fibrosis-4. 

**Figure 1:**
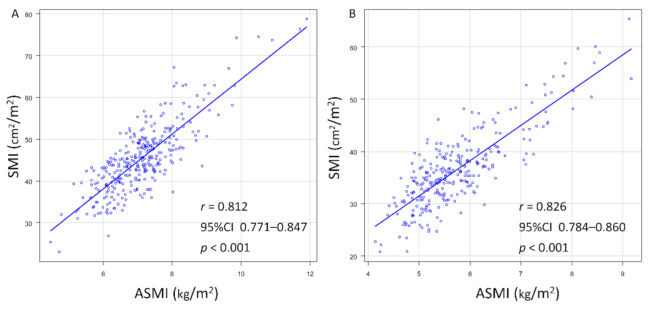
Correlations between the SMI and ASMI in (**A**) men and (**B**) women. ASMI, appendicular skeletal muscle mass index; SMI, skeletal muscle mass index.

We analyzed the correlation between SMI and ASMI in each sex. SMI and ASMI were significantly positively correlated in both sexes (men: *r* = 0.812, 95% CI: 0.771 to 0.847, *p* < 0.001; women: *r* = 0.826, 95% CI: 0.784 to 0.860, *p* < 0.001; Figure 1A,B). 

**Figure 2:**
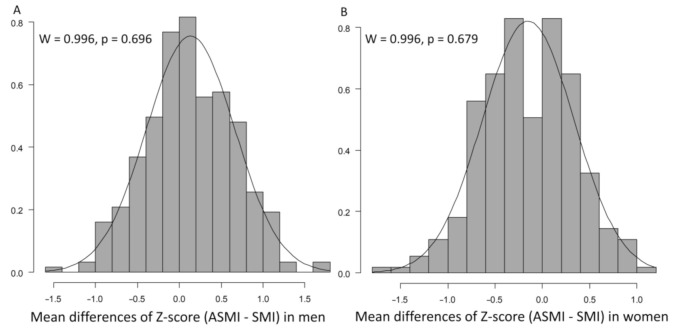
Mean differences of Z-scores (ASMI minus SMI) in (**A**) men and (**B**) women. Normality was examined using the Shapiro–Wilk test. ASMI, appendicular skeletal muscle mass index; SMI, skeletal muscle mass index; *p*, *p*-value.

The mean differences of Z-score (ASMI minus SMI) were normal (Figure 2A,B). 

**Figure 3:**
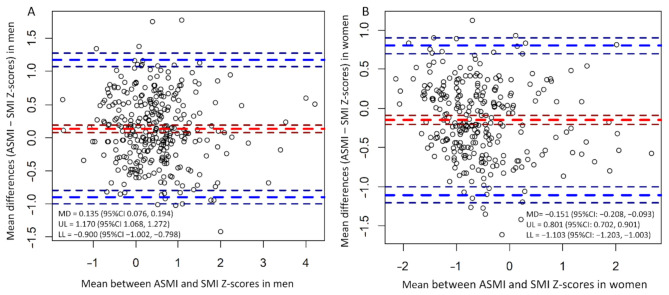
Bland–Altman plot of muscle mass z-scores derived from the skeletal muscle mass index and appendicular skeletal muscle mass index. It represents 95% limits of agreement and 95% CIs between muscle mass z-scores derived from ASMI and SMI in (**A**) men and (**B**) women. ASMI, appendicular skeletal muscle mass index; SMI, skeletal muscle mass index; MD, mean differences; CI, confidence interval; UL, upper limit; LL, lower limit.

Additionally, Bland–Altman plots were constructed to compare the two methods based on their Z-scores (Figure 3A,B). The mean difference (±SD) was 0.135 (0.528) in men and −0.151 (0.486) in women. The upper and lower limits of agreement were 1.170 and −0.900 in men and 0.801 and −1.103 in women. The correlation between mean differences and mean was −0.084 (95% CI: −0.193 to 0.023, *p* = 0.138) in men, and −0.154 (95% CI: −0.267 to −0.037, *p* = 0.010) in women.

**Figure 4:**
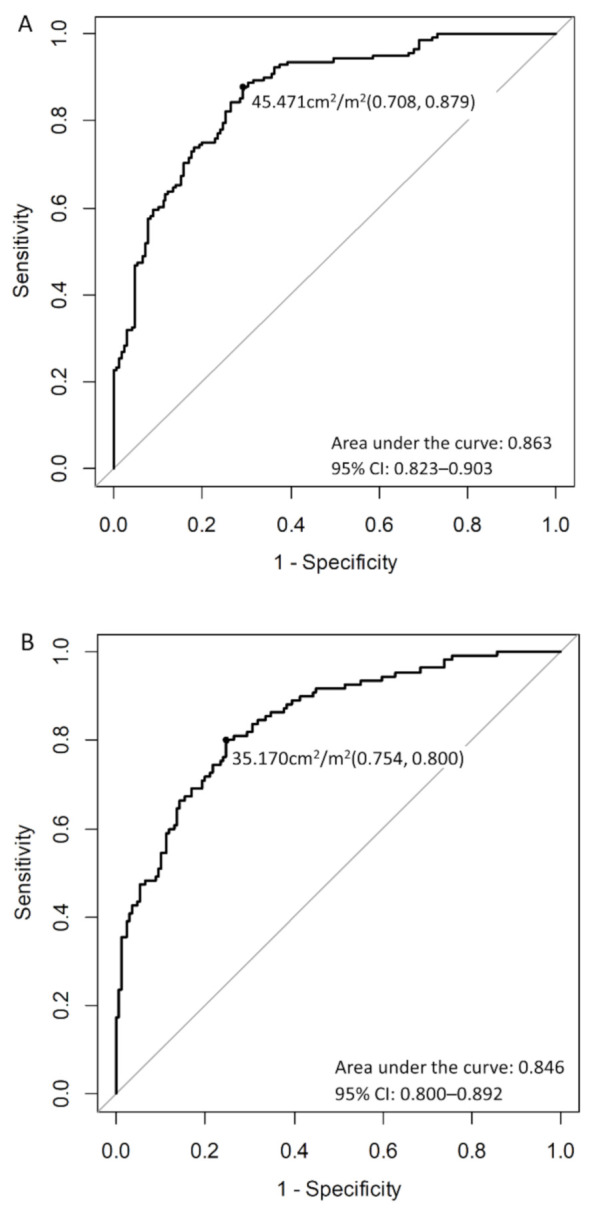
Receiver operating characteristic curves and the cut-off values for (**A**) men and (**B**) women. CI, confidence interval.

Then, the cut-off values of SMI for presarcopenia were calculated by ROC (Figure 4A,B). In men, the cut-off value of SMI for presarcopenia was determined to be 45.471 cm^2^/m^2^, with an area under the curve (AUC) of 0.863 (95% CI: 0.823 to 0.903), and in women, this value was determined to be 35.170 cm^2^/m^2^, with an AUC of 0.846 (95% CI: 0.800 to 0.892).

The diagnostic accuracy of CT in evaluating presarcopenia was 0.785 in men and 0.773 in women. However, Cohen’s kappa coefficient was 0.575 in men and 0.539 in women. There was a large difference in diagnostic accuracy between men and women evaluated by the JSH criteria (Table 2). 

## 4. Discussion

Recently, four methods have been commonly used to assess SMM: CT, BIA, DXA, and magnetic resonance imaging [1,3]. In particular, DXA has been shown to be reliable and valid and is widely used globally [24]. However, there are controversies regarding cost, ease of use, accessibility, and accuracy in each method; each method has strengths and limitations. An accepted threshold for the diagnosis of sarcopenia is required to resolve these issues. The present study enhances the validity of CT-based sarcopenia diagnosis in patients with CLD. Our study proposed a new cut-off point of SMI by CT for presarcopenia in CLD patients. The cut-off point was estimated based on ASMI by DXA and AWGS criteria. The cut-off values were 45.471 cm^2^/m^2^ with an AUC of 0.863 in men, and 35.170 cm^2^/m^2^ with an AUC of 0.846 in women. In Japan, CLD patients undergo CT scans for the evaluation of HCC; therefore, it is possible to simultaneously assess SMM and HCC using CT. The JSH threshold (men: 42.0 cm^2^/m^2^; women: 38.0 cm^2^/m^2^) is widely used in Japan. It is a useful standard created with consideration of the characteristics of liver disease. On the other hand, previous studies of HCC patients have shown different thresholds (men: 36.2 cm^2^/m^2^; women: 29.26 cm^2^/m^2^) [25]. In addition, the L3-SMI cut-off values expressed by Carey et al [26] (men: 50 cm^2^/m^2^; women: 39 cm^2^/m^2^) are closest to the DXA diagnosis in detecting presarcopenia in patients with cirrhosis [14]. Muscle mass is significantly reduced in severe cirrhosis; however, the influence of liver severity may have been limited in this study. Most of the population in this study had cirrhosis though. Therefore, the cut-off values shown in this study were closer to JSH criteria than in other studies.

As stated above, there is not enough consensus on the diagnosis of presarcopenia. Compared to present and previous studies, the JSH criteria do not differ on the threshold between men and women. This suggests that sarcopenia is over-diagnosed or under-diagnosed in either sex. Our results also show a difference in the diagnosis of presarcopenia between AWGS and JSH. In this study, Bland–Altman plots showed that there is a systematic bias in women (*r* = −0154, 95% CI: −0.267 to −0.037, *p* = 0.010). In women, increased muscle mass could increase the difference between ASMI by DXA and SMI by CT. Furthermore, the diagnostic validity in women evaluated using the JSH criteria was low (accuracy: 0.682, kappa: 0.402). Therefore, the diagnostic accuracy of CT in evaluating presarcopenia in women using JSH criteria requires improvement. The difference of measurement sites could have caused the differences in prevalence between the sexes. The AWGS criteria were based on ASM, and the JSH criteria were based on SMM at the L3 level. In recent studies, muscle quality and muscle mass are considered important, and muscle quality in women is lower than in men [27]. Poor muscle quality caused increased mortality after liver transplantation [28]. Women were speculated to have poorer muscle quality than men. Hormones (such as estrogen, testosterone and progesterone) contribute to this difference between genders. In particular, estrogen is a mediator of muscle quality, and the loss of estrogen is associated with a decrease in muscle quality [29]. However, SMI at the L3 level does not evaluate muscle quality. Therefore, further research is needed to improve the reliability and validity of the cut-off values, including muscle mass and quality.

The hand grip strength (HGS) test is quite useful and convenient for sarcopenia screening. Due to the inclusion of the HGS test in the main criteria [1,3,10,30], it is widely used in community-dwelling people and outpatients. It can be carried out at a low cost and without invasion regardless of the facility or location. However, there are issues surrounding the reliability of measuring HGS, such as differences between measuring instruments [31] and the requirements of multiple trials and good posture [1]. In comparison, measurement of muscle mass has the advantage of being objective and reliable. The development of artificial intelligence will improve reliability and accuracy in the measurement of muscle mass [32]. The measurement of SMM has a few limitations, such as high cost, invasiveness, and the need for special equipment. Nevertheless, a previous study revealed that SMM is a predictive factor of prognoses [33,34] and outcomes of treatment [35] in various malignancies. These pathophysiological mechanisms are explained by the myokine. Myokine production is decreased with the loss of SMM [36]. Of the myokines, interleukin (IL)-6 and 15 regulate innate and adaptive immunity systems [37]. Decreased IL-15 caused by low skeletal muscle reduces the number and activity of natural killer cells and worsens the prognosis of cancer patients. In the context of SMM and immune function, the importance of evaluating SMM was shown. 

As has been noted, loss of muscle mass is a predictive factor regarding prognosis and survival in patients with CLD [10]. Since CT is routinely performed as screening for HCC, assessing muscle mass by CT is convenient. Hanai et al. reported that skeletal muscle mass declined by 2.2 per year in patients with LC. According to their results, the decrease in muscle mass becomes more severe as the hepatic condition worsens [7]. CLD patients face a risk of loss of muscle mass. Therefore, assessing muscle mass and diagnosing presarcopenia are feasible and valuable in clinical practice.

Our findings have some limitations and strengths. First, participants in the study consisted of those at various stages of liver disease. Low cut-off values had been shown in patients with HCC [25]. The presence of HCC or LC might cause lower SMM. Thus, it is necessary to consider HCC and hepatic reserves when estimating the cut-off value. Second, our results do not show the prognosis of patients with presarcopenia. Further study is needed to clarify the significance of this new cut-off value. However, a previous cohort study has shown that sarcopenia diagnosed by AWGS had an increased mortality risk [38]. Therefore, the new cut-off values may also predict the prognosis of CLD patients. 

In conclusion, the optimal cut-off values of SMI for presarcopenia were 45.471 cm^2^/m^2^ in men and 35.170 cm^2^/m^2^ in women. SMI by CT showed a strong correlation with ASMI by DXA. However, Cohen’s kappa coefficient was 0.575 (95% CI: 0.485 to 0.665) in men and 0.539 (95% CI: 0.298 to 0.505) in women. Although compatibility of ASMI and SMI was partially agreed, when diagnosing sarcopenia of CLD patients using ASMI or SMI, those differences should be given attention. This study enhanced the validity of CT-based sarcopenia diagnosis in patients with CLD; however, further research is required to make ASMI and SMI fully compatible in patients with CLD.

## Figures and Tables

**Table 1:** Clinical features of patients with chronic liver disease.

Factor	All Patients, *n* = 589	Men, *n* = 312	Women, *n* = 277	*p*-Value
Age, years	64.60 (13.35)	63.15 (13.69)	66.22 (12.79)	0.005
Body mass index, kg/m^2^	23.33 (3.90)	23.62 (3.71)	23.00 (4.10)	0.057
Hepatocellular carcinoma, yes	123 (20.9%)	90 (28.8%)	33 (11.9%)	<0.001
Liver cirrhosis, yes	164 (27.8%)	97 (45.1%)	67 (24.2%)	0.066
Presarcopenia (by AWGS), yes	251 (42.6%)	141 (45.2%)	110 (39.7%)	0.183
Presarcopenia (by JSH), yes	278 (47.2%)	98 (31.4%)	180 (65.0%)	<0.001
Skeletal muscle mass index by CT, cm^2^/m^2^	41.21 (8.94)	45.41(8.22)	36.47(7.18)	<0.001
Skeletal muscle mass index by DXA, cm^2^/m^2^	6.47 (1.17)	7.11(1.02)	5.74(0.88)	<0.001
Whole-body bone mineral density, g/cm^2^	1.00 (0.14)	1.07 (0.12)	0.92 (0.12)	<0.001
Etiology (Virus/Alcohol/NASH/others)	313/55/116/54/51	176/48/57/31	137/7/59/74	<0.001
ALBI score	−2.77 (0.43)	−2.77 (0.45)	−2.77 (0.42)	0.908
ALBI grade (1/2/3)	437/146/6	226/82/4	211/64/2	0.518
FIB-4 index	2.80 (2.62)	2.55 (1.94)	3.07 (3.20)	0.015
Platelet counts, ×10^4^/μL	19.56 (7.99)	19.49 (8.55)	19.64 (7.33)	0.814
Serum albumin, g/dL	4.06 (0.47)	4.07 (0.50)	4.05 (0.41)	0.593
Total bilirubin, mg/dL	1.09(8.59)	0.75(0.52)	1.48(12.51)	0.298
Aspartate aminotransferase, U/L	46.18 (106.20)	38.22 (44.51)	55.04 (147.13)	0.055
Alanine aminotransferase, U/L	46.02 (115.30)	39.19 (47.38)	53.71 (160.25)	0.127

**Table 2:** Validation of each cut-off value for presarcopenia based on CT referenced ASMI by DXA.

	Men (Our Result)	Men (JSH)	Women (Our Result)	Women (JSH)
Prevalence (95% CI)	0.558 (0.501–0.614)	0.314 (0.263–0.369)	0.466 (0.406–0.526)	0.650 (0.590–0.706)
kappa (95% CI)	0.575 (0.485–0.665)	0.515 (0.417–0.612)	0.539 (0.438–0.639)	0.402 (0.298–0.505)
Sensitivity (95% CI)	0.879 (0.814–0.928)	0.589 (0.503–0.671)	0.800 (0.713–0.870)	0.918 (0.850–0.962)
Specificity (95% CI)	0.708 (0.633–0.775)	0.912 (0.859–0.950)	0.754 (0.682–0.818)	0.527 (0.448–0.605)
PPV (95% CI)	0.713 (0.639–0.779)	0.847 (0.760–0.912)	0.682 (0.594–0.761)	0.561 (0.485–0.635)
NPV (95% CI)	0.877 (0.810–0.927)	0.729 (0.664–0.787)	0.851 (0.784–0.904)	0.907 (0.831–0.957)
PLR (95% CI)	3.008 (2.363–3.827)	6.711 (4.059–11.093)	3.259 (2.458–4.319)	1.941 (1.638–2.300)
NLR (95% CI)	0.170 (0.108–0.269)	0.451 (0.368–0.552)	0.265 (0.181–0.389)	0.155 (0.082–0.295)
Accuracy (95% CI)	0.785 (0.735–0.830)	0.766 (0.715–0.812)	0.773 (0.719–0.821)	0.682 (0.624–0.737)

SMI, skeletal muscle mass index; CT, computed tomography; ASMI, appendicular skeletal muscle mass index; DXA, dual-energy X-ray absorptiometry; JSH, Japanese Society of Hepatology; PPV, positive predictive value; NPV, negative predictive value; PLR, positive likelihood ratio; NLR, negative likelihood ratio.

## Data Availability

The data presented in this study are available on request from the corresponding author. The data are not publicly available due to ethical restrictions.
